# Assessing the Therapeutic Role of Rehabilitation Programs in Chemotherapy-Induced Peripheral Neuropathy (CIPN)—A Scoping Review

**DOI:** 10.3390/healthcare13131526

**Published:** 2025-06-26

**Authors:** Yazan A. Al-Ajlouni, Omar Al Ta’ani, Sophia A. Zweig, Magdalena Bak, Mohammad Tanashat, Ahmed Gabr, Zaid Khamis, Farah Al-Bitar, Mohammad Islam

**Affiliations:** 1 School of Medicine, New York Medical College, Valhalla, NY 10595, USA; 2 Department of Physical Medicine and Rehabilitation, Metropolitan Hospital, New York, NY 10029, USA; 3 Department of Medicine, Staten Island University Hospital, Staten Island, NY 10305, USA; 4 Alleghany Health Network, Pittsburgh, PA 15212, USA; 5 School of Medicine, SUNY Downstate College of Medicine, Brooklyn, NY 11203, USA; 6 School of Sciences, New York University Abu Dhabi (NYUAD), Abu Dhabi P.O. Box 129188, United Arab Emirates; 7 Faculty of Medicine, Yarmouk University, Irbid 21163, Jordan; 8 Homer Stryker M.D. School of Medicine, Western Michigan University, Kalamazoo, MI 49007, USA

**Keywords:** chemotherapy-induced peripheral neuropathy, rehabilitation programs, pain management, neuropathic symptoms, scoping review

## Abstract

**Background:** Chemotherapy-induced peripheral neuropathy (CIPN) is a common, debilitating side effect of cancer treatment. Characterized by symptoms like pain, numbness, and muscle weakness, CIPN significantly impacts patients’ quality of life. Current management strategies vary, with limited consensus on effective treatments. This scoping review aims to explore comprehensive rehabilitation interventions for CIPN, focusing on enhancing patient well-being and functional abilities. **Methods:** A scoping review, guided by Arksey and O’Malley’s framework and Levac et al.’s refinements, was conducted to assess rehabilitation programs for CIPN. Searches across six databases were performed, with inclusion and exclusion criteria focusing on studies with physical rehabilitation interventions. Data were charted, detailing interventions, demographics, and outcomes. Results were synthesized descriptively and presented narratively with tables. **Results:** The review included 24 studies covering diverse cancer types and treatments, involving a total of 1167 participants. Various interventions for CIPN were assessed, and results were thematically categorized according to exercise category. Physical modalities like ultrasound and exercise showed promise in symptom relief for colorectal and breast cancer patients. No distinct advantage was found in the timing of exercise interventions. Complementary therapies such as acupuncture and yoga demonstrated effectiveness in managing CIPN symptoms. **Conclusions:** This review highlights the effectiveness of diverse physical and complementary interventions in managing CIPN, advocating for their integration into standard protocols. It emphasizes the need for holistic, patient-centered approaches that combine exercises, physical therapy, and complementary therapies to improve patient outcomes. These findings set a direction for future research and clinical practices focused on comprehensive and personalized CIPN management strategies.

## 1. Introduction

Chemotherapy-induced peripheral neuropathy (CIPN) stands as a significant and often debilitating side effect of cancer treatment, casting a shadow over the lives of countless patients battling this formidable disease. As a neurological disorder triggered by the administration of chemotherapeutic agents, especially those that are platinum-based (e.g., Cisplatin), CIPN encompasses a wide spectrum of symptoms, including tingling sensations, numbness, pain, and muscle weakness, which typically manifest in the hands and feet [1]. Several conditions may be associated with CIPN including autonomic dysfunction, sleep disturbances, fatigue, and mood disorders such as anxiety and depression [2]. These conditions can worsen the functional impairments and can significantly reduce quality of life. CIPN follows a glove-and-stocking pattern with symmetric loss of sensation in distal extremities. Symptoms may persist in some cases after the completion of chemotherapy, causing long-term disability, gait instability, and increased risk of falls [3].

The prevalence of CIPN among patients receiving chemotherapy varies widely, with estimates ranging from 12% to 96% [2,4,5], depending on the specific drugs and dosages administered [6]. Beyond the physical discomfort it inflicts, CIPN holds profound implications for the overall well-being and treatment outcomes of individuals already grappling with the burdens of cancer [7,8]. This variability in CIPN’s impact stresses the need for effective management strategies to address the wide-ranging challenges it poses to patients.

To date, the evidence regarding CIPN’s management continues to be inconsistent, with some reports concluding that neuropathies cannot be treated, and protective treatment strategies have not been significantly effective [9]. Other reports have suggested positive treatment efforts, including decompression surgery [10] and pharmacological strategies [11,12]. Furthermore, emerging research in the literature has explored the role of exercise in the management of CIPN’s symptoms. For instance, previous systematic reviews reported significant improvements in postural control, quality of life, muscular strength, and independence [13,14].

Furthermore, CIPN’s symptoms extend beyond physical manifestations to an emotional toll on patients, especially as they fear compromised treatment efficacy. Consequently, this makes it imperative to address CIPN using a comprehensive approach, one that not only addresses the physical symptoms but also seeks to enhance patients’ overall quality of life. In this context, emerging strategies within the field of Physical Medicine and Rehabilitation (PM&R) offer promising avenues for improving the well-being of cancer patients undergoing chemotherapy. PM&R, as a specialty that primarily focuses on enhancing patients’ functional abilities and quality of life, may hold the key to effective CIPN management by providing holistic interventions that extend beyond pharmacological or surgical approaches. To date, *and to the best of our knowledge*, there has been limited exploration in the literature reviewing the evidence of comprehensive rehabilitation approaches for CIPN. Previous research has predominantly focused on exercise programs and behavioral interventions [15,16]. However, considering the increasing attention to the long-term effects of CIPN and the potential impact of rehabilitation on improving patients’ functions and quality of life [17], a scoping review of the extent of multidisciplinary rehabilitation programs on CIPN could significantly enhance our understanding in this domain.

In light of these considerations, this study aimed to conduct a scoping review to explore the breadth and depth of comprehensive rehabilitation interventions for CIPN. By encompassing a wider range of rehabilitation interventions, this scoping review aims to provide a more holistic view of the strategies employed to address CIPN. The insights gained from this review are expected to inform clinical practice and aid in the development of tailored rehabilitation interventions that meet the specific needs of patients with CIPN. This is particularly pertinent given the global expansion of PM&R, a field inherently multidisciplinary in nature, emphasizing a holistic approach to patient care that integrates various therapeutic modalities to optimize function and quality of life.

## 2. Methods

A scoping review was conducted following the methodological framework suggested by Arksey and O’Malley [18] and further refined by Levac et al. [19]. This approach was chosen instead of PRISMA-ScR to explore the breadth of literature and map the key concepts and evidence concerning rehabilitation programs on CIPN. This review was not registered on a platform such as the Open Science Framework, as its primary goal was exploratory conceptual mapping rather than hypothesis testing—a common practice for Arksey and O’Malley-based scoping reviews.

**Figure 1:**
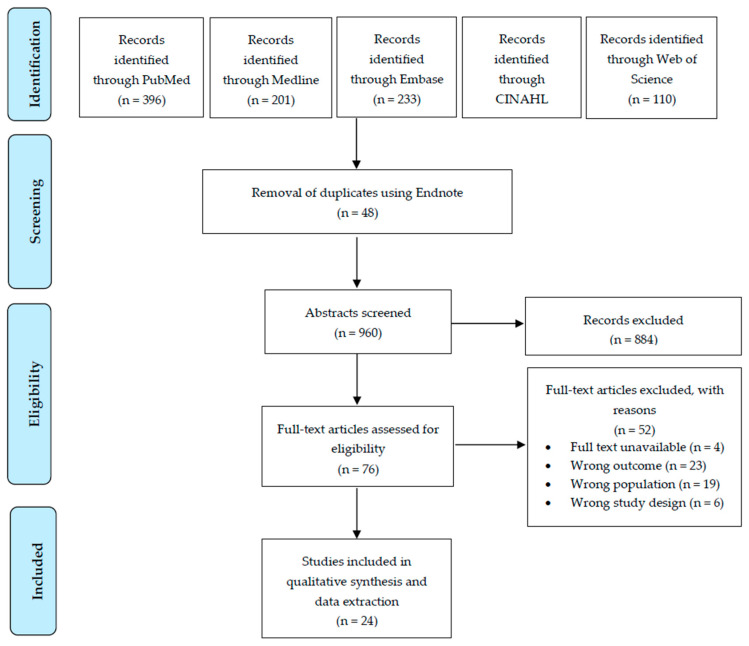
Preferred Reporting Items for Systematic Reviews and Meta-Analyses (PRISMA) study selection flow diagram outlining the literature review process when searching for articles on various databases.

While PRISMA-ScR is a valuable tool for structured reporting [20], our review adopted the Arksey and O’Malley framework [18], refined by Levac et al. [19], as it better aligns with our exploratory aims to map broad evidence rather than answer a narrow clinical question. This approach emphasizes iterative literature engagement and conceptual mapping, which was critical given the heterogeneity of CIPN rehabilitation interventions. However, to enhance transparency, we now include a PRISMA-ScR checklist (Table S1) and flow diagram (Figure 1).

### 2.1. Search Strategy and Selection Criteria

This study sought to examine what is known about the effects of rehabilitation programs on CIPN from the existing literature. A comprehensive search strategy was designed using a set of developed keywords (outlined in Table S2 in the Supplementary File). Searches were conducted across multiple databases: (1) PubMed; (2) PsycINFO; (3) Web of Science; (4) EMBASE; (5) ScienceDirect; (6) CINAHL. Boolean operators were used to enhance the search.

Initial search results (n = 1008) were managed using Endnote reference manager software (Endnote X9.3.3). After duplicate removal (n = 48), titles and abstracts of unique search results (n = 960) were screened for relevance by two independent reviewers (Y.A.A and M.T). The inclusion and exclusion criteria were iteratively developed and refined during the screening process, with emphasis on studies involving human subjects, published in English, and focusing on physical rehabilitation interventions for patients with CIPN. Table S3 in the supplementary file details the complete eligibility criteria post-screening.

### 2.2. Data Charting

Data charting was performed to identify and outline the breadth and variety of studies in the domain of rehabilitation programs for CIPN. A data charting form was developed and employed to systematically extract and organize key information from each study.

The parameters extracted included a detailed description of the physical intervention along with its duration. Additionally, information about the comparator group or any other intervention was noted, including a full description and the duration of these interventions. The sample size of each study was recorded, as well as demographic data such as mean age (with standard deviation), gender distribution (expressed as number and percentage), and the cause of neuropathy. Further details were charted about the type of chemotherapy used, the cancer types treated, and any other treatments administered alongside the primary intervention.

A crucial aspect of charting involved documenting the signs of neuropathy as reported in each study, providing insights into the clinical presentation of CIPN in the context of the rehabilitation programs. Lastly, the main findings of each study were extracted, focusing particularly on the outcomes and effectiveness of the rehabilitation interventions compared to the comparators.

This meticulous process of data charting was initiated by a single reviewer and was subsequently validated by the coauthors to ensure accuracy and comprehensiveness. The data collected offers a panoramic view of the current research landscape on rehabilitation interventions for CIPN, highlighting varied approaches, methodologies, and findings across different studies.

### 2.3. Data Presentation and Analysis

Data were synthesized descriptively, focusing on mapping the extent, range, and nature of the research activity in this field. The results were presented in a tabular format and accompanied by a narrative summary that highlights key trends, demographic characteristics, types of interventions, and reported outcomes in the studies reviewed.

## 3. Results

The identified studies examined various physical interventions for the treatment of CIPN. Studies by Al Onazi et al. and Hammond et al. delve into physical modalities, with ultrasound therapy exhibiting promise for colorectal cancer patients and home exercises showing potential benefits for those with breast cancer. Bland et al. explored exercise timing, revealing no distinct advantages in immediate versus delayed supervised training for breast cancer patients. Complementary therapies, such as acupuncture, demonstrated effectiveness, suggesting a role in managing CIPN symptoms. Furthermore, yoga interventions [21] and transcutaneous electrical nerve stimulation [22] displayed positive outcomes.

### 3.1. Overview of Studies Included

The review consists of 24 studies published from 2019 to 2023. Geographically, the studies cover diverse regions: Canada [23,24], the USA [22,25,26], Sweden [27], Israel [28], China [29], Iran [29], Japan [30], Turkey [31], Germany [32,33], Republic of Korea [34], and India [35]. The reviewed papers examine multiple cancer types, including colorectal [24,29,36,37,38], breast [23,28,29,31,36,39,40,41], as well as many others like lung, ovarian, prostate, colorectal, colon, rectum, gynecological, and lymphoma [26,29,36,41]. Diverse chemotherapy medications were used, with the most prominent being oxaliplatin [21,22,24,30,38,41] and paclitaxel [22,25,28,30,31,34,35,39,41,42,43]. The characteristics of the included studies are summarized in Table 1.

### 3.2. Comparators Used in Studies

The studies included in the review employed diverse control interventions and standard care approaches for comparison. Common control interventions involved a placebo [34,36] or sham treatment [39] to assess the specific effects of the intervention under investigation. Additionally, some studies compared the intervention against standard care practices [23,24,28,41] ensuring a benchmark for evaluating its efficacy.

### 3.3. Types and Efficacy of Interventions

**Figure 2:**
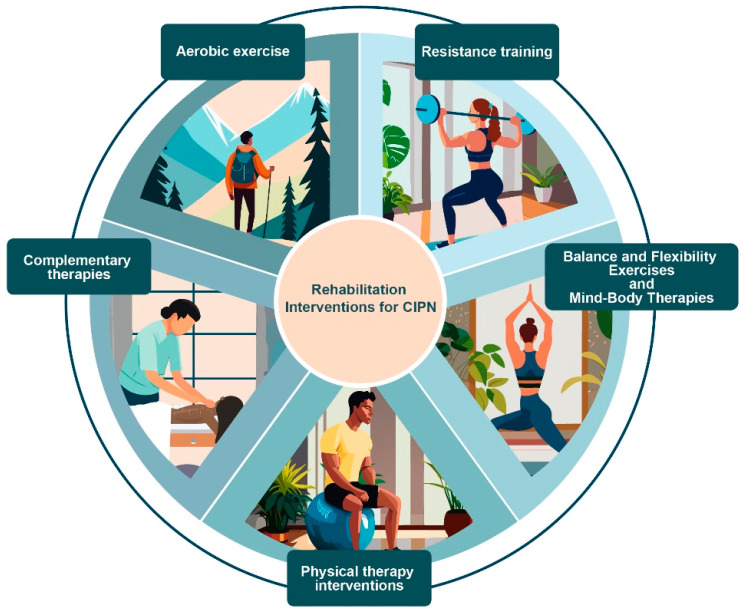
Different categories of rehabilitation interventions for chemotherapy-induced peripheral neuropathy identified in the scoping review.

Figure 2 illustrates the various interventions identified in this scoping review, as described in detail in this subsection.

#### 3.3.1. Aerobic Exercise

Bland et al. [43] studied the impact of immediate versus delayed exercise (IE vs. DE), including physical intervention (aerobic exercise) such as immediate exercise (supervised aerobic, resistance, and balance training), which was offered 3 days a week for 8–12 weeks. Although the exercise interventions showed no significant differences in managing CIPN, both groups experienced increased sensory and motor symptoms during chemotherapy, with recovery post-treatment, and the IE group reported a lower percentage of participants with numbness in the toes or feet at pre-cycle 4 compared to the DE group.

#### 3.3.2. Resistance Training

In examining the effectiveness of resistance training as an intervention for CIPN, several studies have presented compelling evidence. Streckmann et al. and Zimmer et al. both highlighted the benefits of supervised exercise programs, which encompassed resistance training among other activities [32,33,38,43].

Streckmann et al. conducted two pivotal studies, each exploring different facets of exercise interventions. In their first study [33], the intervention group underwent supervised exercise twice a week for 1 h each over 36 weeks, incorporating activities like cycling or treadmill walking, postural stabilization tasks, and resistance exercises. The control group continued chemotherapy without structured exercise. The intervention group showed significant improvements in quality of life, constipation, diarrhea, and pain, with a lower incidence of peripheral neuropathy (PNP) compared to the control group. The intervention group also exhibited increased activity levels and improved balance control. In Streckmann et al.’s other study [32], participants engaged in supervised training twice a week for 6 weeks, with two intervention arms: sensorimotor training (SMT) and whole-body vibration training (WBV). SMT involved progressively challenging balancing exercises, while WBV utilized a vibration platform. The SMT group demonstrated improved Achilles and patellar tendon reflexes compared to the control group, with reduced pain and dyspnea. The WBV group also showed improvements in pain and dyspnea. 

Adding to the body of evidence on the topic, Zimmer et al. [38] conducted an eight-week supervised exercise program for individuals who had undergone chemotherapy. The program included endurance, resistance, and balance training conducted twice weekly for 60 min each session. The control group received written standard recommendations to achieve physical fitness. The participants had colorectal cancer and were exposed to oxaliplatin-based chemotherapy. The exercise group exhibited improved physical fitness and quality of life compared to the control group, which received only written standard recommendations for physical activity.

#### 3.3.3. Balance and Flexibility Exercises and Mind–Body Therapies

This subsection explores the promising role of balance, flexibility exercise, and mind–body therapies. These exercises encompass practices like yoga, tai chi, qigong, and mindfulness techniques. They aim to enhance both physical and mental well-being through a combination of physical postures, controlled breathing, and meditation.

In a study by Galantino et al. [27], cancer survivors with CIPN participated in somatic yoga and meditation (SYM) sessions twice a week for 8 weeks. These 90 min sessions, led by certified yoga instructors, aimed to address the sensory and motor deficits associated with CIPN. Somatic movements use pandiculation, voluntary muscular contraction, and slow, controlled decontraction (eccentric contraction), with a constant focus on sensation, to increase the resting length of muscles. The study found that SYM had positive effects on both clinical measurements and patient-reported symptoms, indicating its potential in improving proprioceptive signals, enhancing postural control, and reducing the risk of falls.

Similarly, Knoerl et al. [21] conducted research involving 28 individuals with CIPN, predominantly comprising older women who had undergone various chemotherapy regimens. The participants engaged in at least 12 modified Hatha yoga sessions over 8 weeks. The study reported that yoga brought about improvements in well-being across different cancer types and chemotherapy regimens.

Zhi et al. [25] further contributed to this body of research by examining the effects of an 8-week yoga intervention on individuals who had completed neurotoxic chemotherapy. Participants engaged in daily 60 min yoga sessions, which included breathwork (pranayama) and adaptable postures (asanas). The yoga group showed significant reductions in pain, improvements in neuropathy-specific quality of life (FACT/GOG-Ntx scores), and enhanced functional reach, which is crucial in predicting fall risk.

Furthermore, Ben-Arye et al. [28] investigated the impact of complementary integrative medicine (CIM) on patients with breast or gynecological cancer undergoing taxane-based chemotherapy or those with hematological malignancies receiving neurotoxic treatments. In the intervention arm, patients received twice-weekly CIM sessions for 6 weeks, including acupuncture and other manual or mind–body therapies. During the baseline-to-6-week assessments, participants in the intervention group experienced notable improvements in emotional well-being and overall FACT-Tax scores compared to the control group. Specifically, they reported reduced hand numbness/tingling and discomfort, along with enhanced physical functioning as measured by the EORTC scale. Additionally, both intervention groups (A and B) showed significant enhancements in various aspects of quality of life, including physical well-being, total FACT-Tax score, and reduction in feet discomfort. Moreover, there were improvements in EORTC pain scores.

#### 3.3.4. Physical Therapy Interventions

This subsection delves into the impact of physical therapy interventions on CIPN, drawing from studies by Ikio et al. and Al Onazi et al. [24,30].

Ikio et al. [30] focused on muscle strength exercises, manual dexterity training, and sensory function training among patients with hematological or gastrointestinal malignancies. The intervention group demonstrated a substantially lesser decline in activities of daily living compared to the control group at the second time point (defined in the trial as two chemotherapy cycles), as measured by the Michigan Hand Outcomes Questionnaire (MHQ). This was evident in both intention-to-treat and as-treated analyses, with notable improvements in pain management.

Al Onazi et al. [24] explored the combination of ultrasound therapy (3 cm transducer, 3 MHz frequency, and continuous ultrasound at an intensity of 0.7–0.8 w/cm^2^ for 5 min to each location), with a home exercise regimen in colorectal cancer patients experiencing chemotherapy-induced hand and foot pain. Over the first two weeks, participants underwent 10 sessions of ultrasound therapy, applying a 3 cm transducer with a frequency of 3 MHz and continuous ultrasound at an intensity of 0.7 to 0.8 w/cm² for 5 min to each location (fingers and toes/base of feet). The standard care group followed a 6-week regimen comprising education on CIPN, a home exercise program, and education on self-care strategies.

#### 3.3.5. Complementary Therapies

The exploration of complementary therapies in the treatment of CIPN has gained notable attention, as seen in studies by Iravani et al., Greenlee et al., and Izgu et al. [29,31,39]. These research efforts delve into the potential of acupuncture, electro-acupuncture, and classical massage as viable treatment options.

Iravani et al. [29] examined the effectiveness of acupuncture for CIPN among a group of nineteen participants predominantly diagnosed with breast and colorectal cancer. The study found that acupuncture not only safely treated CIPN but also showed superior efficacy compared to conventional treatments such as vitamin B1 and gabapentin.

Greenlee et al. [39] conducted a study to assess the impact of electro-acupuncture (EA) on CIPN in patients with stage I–III breast cancer receiving paclitaxel. This study included 63 participants and used a sham electro-acupuncture (SEA) group as a control. Each group received their respective treatments over a 12-week period. However, patients in the EA group reported worse peripheral neuropathy symptoms than those in the SEA group.

Lastly, Izgu et al. [31] explored the role of classical massage therapy in managing paclitaxel-induced CIPN, particularly in breast cancer patients. Over 12 weeks, nineteen participants received massage sessions aimed at reducing neuropathic pain and enhancing quality of life (QOL). The results showed significant reductions in neuropathic pain and improvements in both sensory and motor QOL sub-scale scores for the massage group. Additionally, the sensory action potential amplitude of the median nerve was significantly higher, and the tibial nerve latency was significantly shorter in the CMG compared to the CG at week 12.

## 4. Discussion

This scoping review explores various physical interventions for managing CIPN, a prevalent and debilitating side effect among cancer patients. Encompassing a diverse array of modalities—from aerobic and resistance exercises to mind–body therapies and tailored physical therapy interventions—this review synthesizes findings from studies conducted across various global contexts. Its primary objective was to evaluate the efficacy and applicability of these interventions in alleviating CIPN symptoms across different cancer types and chemotherapy regimens, with a special focus on prevalent drugs like oxaliplatin and paclitaxel. Our review not only highlights key outcomes such as enhanced quality of life, effective pain management, improved physical fitness, and reduced neuropathy symptoms but also sheds light on the long-term benefits and sustainability of these interventions, thereby offering a comprehensive perspective on potential therapeutic strategies for CIPN.

The integration of physical exercises, particularly aerobic and resistance training, into the management of CIPN suggests potential benefits. The results of studies examining these interventions [32,33,38,43] indicate that these forms of exercise may play a role in both alleviating existing CIPN symptoms and preventing their severity. Aerobic exercises have been associated with improvements in overall patient well-being [44,45], while resistance training may enhance muscle strength and functional capacity [46,47]. These studies collectively underscore the pivotal role of structured, supervised physical exercise regimens in managing CIPN. They demonstrate that a diverse range of physical activities, tailored to the needs of chemotherapy patients, can substantially improve not only their physical well-being but also their overall quality of life. This evidence advocates for the integration of such exercise programs into standard care protocols for CIPN management, emphasizing the need for personalized, patient-centered approaches in oncology care. This evidence suggests that a combined approach of both aerobic and resistance exercises might offer a more holistic management strategy for CIPN.

The impact of the timing of these exercise interventions also emerges as an area of interest [43]. Despite some inconclusive results, initiating exercise programs early in the chemotherapy process could influence the development and severity of CIPN. This hypothesis aligns with current research indicating the importance of early intervention in managing chronic conditions [48,49]. Moreover, the focus on supervised training underscores the need for personalized and safe exercise regimens. Supervised exercises ensure patient safety [50,51] and could optimize therapeutic outcomes, possibly leading to improvements in neuropathy symptoms and quality of life. These insights, while preliminary, suggest the potential for integrating structured exercise into standard CIPN management protocols.

Furthermore, balance, flexibility exercises, and mind–body therapies, such as yoga, have emerged as promising complementary approaches. The outcomes of these interventions point toward notable improvements in proprioception and balance, which are critical for patients experiencing the disorienting effects of CIPN. Additionally, these therapies have shown effectiveness in managing pain, a primary concern for many undergoing chemotherapies [52]. Yoga has an integrative approach, which utilizes a combination of physical movement, breathing exercises, and mindfulness and has shown promising results among mind–body therapies. Gentle yoga for CIPN patients focuses on balance, stretching, and relaxation, which can be adapted to accommodate sensory deficits and muscle weakness. Studies have shown that yoga improves pain, balance, fatigue, and psychological outcomes among cancer survivors, including those with CIPN [53,54]. It is imperative to consider the implementation of such practices under expert supervision, especially in oncology, to ensure safety and efficacy.

By improving sensory and motor functions, reducing pain, and enhancing quality of life, these non-pharmacological approaches offer valuable complementary strategies for CIPN management in cancer survivors. Their adaptability across various cancer types and chemotherapy regimens further underscores the potential of incorporating these practices into holistic care plans for individuals suffering from CIPN. Beyond physical benefits, these practices have a profound impact on the overall quality of life, offering a holistic approach to managing the multifaceted challenges of CIPN. The therapeutic value of these interventions, as suggested by current research, lies not only in their physical aspects but also in their ability to address the psychological and emotional strains faced by patients [55,56,57]. This dual impact on both physical and mental health positions these therapies as valuable components of comprehensive CIPN management strategies.

Physical therapy interventions have shown promise in the management of CIPN, offering a range of techniques tailored to individual patient needs. These interventions, ranging from specific exercises and ultrasound therapy to comprehensive approaches that integrate acupuncture and exercise, demonstrate substantial promise in improving patient experiences. Among these, muscle strength exercises and sensory function training stand out. While muscle strength exercises focus on the physical debilitations of CIPN, sensory function training addresses the nuanced sensory deficits, aiding in the re-establishment of proprioception and coordination, enhancing functionality, and improving daily living activities [24,30]. Their effectiveness is evident in enhancing daily activities, alleviating pain, and elevating the overall quality of life for individuals undergoing chemotherapy. These studies collectively reinforce the importance and potential of physical therapy as a critical component in the management of chemotherapy-induced complications.

Additionally, the emerging role of ultrasound therapy in CIPN symptom management represents an innovative and increasingly popular approach. Its non-invasive nature offers potential benefits in pain relief and nerve regeneration, although the underlying mechanisms are still under investigation. These sessions often include a variety of therapeutic practices targeting both physical and psychological aspects of CIPN. Thus, the combined use of traditional physical therapy approaches with innovative techniques like ultrasound therapy and CIM sessions demonstrates the evolving landscape of CIPN management, acknowledging the need for multifaceted treatment strategies.

Finally, our review has also shown that complementary therapies, notably acupuncture and massage therapy, are gaining traction in the management of CIPN, as highlighted in recent studies. Acupuncture, with roots in traditional Chinese medicine, has shown promise in reducing CIPN symptoms, potentially offering an alternative for patients who have limited relief from traditional Western treatments. Its efficacy in alleviating pain, tingling, and numbness, as evidenced by Iravani et al., positions it as a valuable option in the CIPN treatment arsenal. Similarly, massage therapy, as explored by Greenlee et al. and Izgu et al. [31,39], has been associated with not only symptomatic relief but also improvements in overall patient well-being. While acupuncture and massage show potential benefits, the varied outcomes with electro-acupuncture indicate a complex landscape, necessitating further research to fully understand and optimize these therapies for CIPN management. The safety profiles of both acupuncture and massage therapy add to their appeal, presenting minimal risks when conducted by trained professionals. The implications of these findings are significant for current treatment practices; they further highlight the need for a more integrative approach to patient care, embracing complementary therapies as part of a holistic treatment plan. This shift not only aligns with patient preferences for non-pharmacological interventions but also addresses a broader spectrum of CIPN symptoms, thereby enhancing the quality of life for cancer survivors. Collectively, these studies illuminate the promising role of complementary therapies in managing CIPN, offering alternative or adjunctive options to traditional medical treatments.

### 4.1. Clinical Implications

The findings of this scoping review emphasize the necessity of integrating diverse physical interventions, including aerobic and resistance exercises, balance and flexibility exercises, and mind–body therapies such as yoga, into the standard care protocols for CIPN. These interventions, tailored to individual patient needs and conditions, hold significant promise in not only alleviating CIPN symptoms but also in enhancing overall patient well-being. There is a critical need for healthcare professionals to be educated and trained in these varied techniques to ensure safe and effective administration. Furthermore, early intervention strategies, particularly for patients at high risk or in the initial stages of chemotherapy, could potentially mitigate the severity of CIPN symptoms. This proactive approach, coupled with patient education and involvement in treatment decisions, may lead to better adherence to therapy and improved outcomes.

Additionally, the growing evidence supporting complementary therapies like acupuncture and massage therapy in CIPN management suggests that these should be more widely integrated into treatment plans, especially for patients seeking non-pharmacological options. These treatment options are often not covered by insurance [58,59,60]; thus, healthcare systems and insurance policies would need to adapt to include these therapies to make them more accessible and affordable. By embracing these clinical implications and strategies, healthcare providers can significantly enhance the quality of life and care for patients suffering from CIPN, marking a pivotal shift in the current management paradigm of this challenging condition.

### 4.2. Limitations and Future Research

This review has several limitations. Firstly, the nature of a scoping review may limit the depth of analysis for each specific intervention. This approach, while beneficial for mapping the range of evidence, does not provide the detailed assessment of quality and robustness that systematic reviews offer. Variations in study designs across the reviewed research introduce challenges in synthesizing and comparing findings, potentially affecting the robustness of our conclusions. Many studies reported general quality-of-life improvements without CIPN-specific quantitative metrics (e.g., neuropathy scales or pain scores), limiting our ability to assess direct impacts on neuropathy symptoms. Moreover, the notable heterogeneity in the types of intervention, outcomes, and patient characteristics among included studies makes it arduous to reach a definitive conclusion regarding the effectiveness of specific interventions. While several interventions have reported desirable outcomes, these should be interpreted with caution. Additionally, the diversity in sample sizes, which ranged from small pilot studies to larger trials, may influence the generalizability of the results. The geographic distribution of the studies also presents a limitation, as the majority are concentrated in certain regions, potentially limiting applicability across diverse populations and healthcare systems.

Future research should focus on addressing these gaps by conducting studies with uniform design methodologies and larger, more diverse sample populations. This would enable more definitive conclusions about the efficacy and applicability of various interventions for CIPN. Further, research efforts could benefit from a more focused approach, such as systematic reviews or meta-analyses, to provide a deeper understanding of specific interventions. Expanding research to include a wider geographic spread can ensure that findings are relevant and adaptable globally. Investigating the long-term effects of these interventions and their impact on the progression of CIPN symptoms would also be valuable. Additionally, exploring the underlying mechanisms through which these therapies exert their benefits could offer insights into novel treatment approaches and refine existing ones for better patient outcomes.

## 5. Conclusions

In conclusion, this scoping review has illuminated the diverse landscape of physical and complementary interventions for managing CIPN, revealing significant potential in improving patient outcomes. Our comprehensive analysis highlights that incorporating a multifaceted approach, encompassing aerobic and resistance exercises, tailored physical therapy, mind–body practices, and emerging complementary therapies like acupuncture and massage therapy, can effectively alleviate CIPN symptoms and enhance the overall quality of life for cancer patients. These findings advocate for a paradigm shift in CIPN management, urging the integration of these varied interventions into standard care protocols and patient-specific treatment plans. As the landscape of CIPN management continues to evolve, it becomes imperative for healthcare systems to adapt, prioritizing patient-centered, holistic care approaches that address both the physical and psychological aspects of this challenging condition. This review paves the way for future research and clinical practice to focus on more comprehensive, personalized, and proactive strategies in the battle against the debilitating effects of CIPN.

## Figures and Tables

**Table 1:** Baseline characteristics of all included studies.

Study ID	PI *	C (Control or Any Other Intervention) *	Sample Size		Age, Mean (SD)	Gender, n (%)			Cause of Neuropathy		Chemotherapy Used		Cancer Type		Othe Treatments		Signs of Neuropathy		Main Findings
			PI	C	PI	C	PI	C	PI	C	PI	C	PI	C	PI	C	PI	C	
Al Onazi et al. 2021 [24]	10 sessions of US therapy over the first two-week period (Monday to Friday) of the study. US was applied to the fingers and toes/base of feet using the following parameters: 3 cm transducer (large sound head), frequency of 3 MHz, and continuous ultrasound at an intensity of 0.7 to 0.8 w/cm^2^ for 5 min to each location.	Standard care (6 weeks)	16	15	59.69	60.57	6 (37.5)	6 (40)	Chemotherapy		Chemotherapy is used in the treatment of colorectal cancer		CRC		5-FU, capecitabine, irinotecan, and oxaliplatin		Pain and sensory disturbance in the hands and feet		The results of this proof-of-concept study demonstrate the viability of therapeutic US plus a home exercise regimen as an intervention for colorectal cancer patients with pain and related symptoms in the hands and feet. Our research design and intervention procedures appear to be practical, based on our 84% recruitment rate and 100% intervention and study completion rates.
Hammond et al. 2020 [23]	The IG was given a home exercise and education program at the start of chemotherapy. The crux of the home program was nerve gliding exercises, which were repeated 3× daily and took 5–10 min to complete. There was instruction on how to handle neuropathic pain, cold intolerance, and hyperalgesia symptoms. The physical therapist only made one follow-up phone call (6 weeks later).	Standard care for the CG	22	26	56.3	53	N/A	N/A	Chemotherapy		Adjuvant taxane chemotherapy for stage I–III patients with breast cancer		Breast cancer		Taxane chemotherapy (as adjuvant treatment). Docetaxel 75 mg/m^2^ and cyclophosphamide 600 mg/m^2^ every 21 days 4 cycles (TC) or (2) 5-FU 500 mg/m^2^, epirubicin 100 mg/m^2^, cyclophosphamide 500 mg/m^2^ given every 21 days 3 cycles, followed by docetaxel 100 mg/m^2^ given every 21 days 3 cycles		Neuropathic pain, cold intolerance, and hyperalgesia		Physical therapy at home may help patients with breast cancer with CIPN discomfort in the upper extremity, and general activity during chemotherapy treatment has been linked to sensory function preservation.
Bland et al. 2019 [43]	Immediate Exercise (Supervised aerobic, resistance, and balance training was offered 3 days a week for 8–12 weeks)	Delayed exercise (Supervised aerobic, resistance, and balance training was offered 3 days a week for 8–12 weeks)	12	15	51	49.5	N/A	N/A	Chemotherapy		Adjuvant taxane chemotherapy for stage I–III patients with breast cancer		Breast cancer		Paclitaxel or docetaxel chemotherapy		N/A		
Dhawan et al. 2020 [35]	Exercise group: home-based muscle strengthening and balancing exercise (10 weeks)	Usual care	22	23	50.5	52.5	4 (18.1)	3 (13.1)	Chemotherapy		Chemotherapy is used in the treatment of any type of cancer		Any type of cancer		Paclitaxel and carboplatin		Altered cotton wool sensations, weakness in lower limbs, tingling in feet, weakness in upper limbs, pins and needles sensations, numbness in feet, tingling in hands, burning sensations, problems of maintaining balance, and sensitivity to cold temperature		
Galantino et al. 2019 [27]	Somatic Yoga and Meditation (Participants met twice per week for 8 weeks, and sessions were taught by certified yoga instructors. The yoga program lasted 90 min per class)	N/A	10	N/A	64.4	N/A	1 (10)	N/A	Chemotherapy		Chemotherapy is used in the treatment of any type of cancer		Any type of cancer		N/A		Sensory (eg, tingling, pain, and numbness) and motor (eg, weakness, walking, eating)		Cancer survivors with CIPN have sensory and motor deficits, which result in improper proprioceptive signals, decreased postural control, and a higher risk of falling. In cancer survivors with CIPN, SYM may improve clinical measurements and patient-reported symptoms.
Greenlee et al. 2017 [39]	Electro-acupuncture (receive 12 weeks of weekly EA)	Sham electro-acupuncture (receive 12 weeks of weekly SEA)	31	32	51.8	48.3	N/A	N/A	Chemotherapy		Stage I–III breast cancer scheduled to receive 12 weeks of weekly adjuvant or neo-adjuvant paclitaxel		Breast cancer		Adjuvant or neo-adjuvant paclitaxel		Numbness in the hands and feet		Patients in the EA arm had worse peripheral neuropathy symptoms than those in the SEA arm.
Ben-Arye et al. 2022 [28]	Intervention arm (groups A and B): Patients choosing to undergo the twice-weekly CIM intervention for 6 weeks, in addition to standard conventional care. Arm were randomized to twice-weekly acupuncture-only (group A) or acupuncture with additional manual-movement or mind–body CIM therapies (group B)	Patients choosing only standard conventional supportive care.	136	32	57.9	56.7	N/A	N/A	Chemotherapy		Female patients with breast or gynecological cancer receiving taxane-based chemotherapy (e.g., paclitaxel, docetaxel). Patients of either sex with hematological malignancies receiving neurotoxic treatment (for example, bortezomib for multiple myeloma).		Breast, gynecological cancer and hematological malignancies		Chemotherapy with taxane-based agents (e.g., paclitaxel, docetaxel) and treated with neurotoxic chemotherapy agents (e.g., Bortezomib for multiple myeloma).		Hand and feet numbness, tingling, pain and discomfort		We discovered that exercise may help to slow the course of CIPN symptoms and maintain qol while allowing more patients to acquire an acceptable taxane RDI.
Ikio et al. 2022 [30]	Muscle strength exercises, manual dexterity training, and sensory function training. Muscle exercises included grip and pinching movements, sensory function training was done through material identification using different surfaces and materials, and tactile perception practice with counted dot numbers using Braille practice sheets. For manual dexterity training, origami and paper tearing were performed. The participants were instructed to perform the program for approximately 30 min a day for ≥3 days/week		19	20	72 (range, 66–89). NOTE: No SD, just range reported	63 (range, 57–87). NOTE: No SD, just range reported	11 (57.9) males, 8 (42.1) females	11 (55.0) males, 9 (45.0) females	Chemotherapy-induced	Chemotherapy-induced	11 (57.9%) Vincristine, 6 (31.6%) Oxaliplatin, 2 (10.5%) Nab-paclitaxel	11 (55.0%) Vincristine, 6 (30.0%) Oxaliplatin, 3 (15.0%) Nab-paclitaxel	11 (57.9%) hematologic, 8 (42.1) GI	11 (55.0%) hematologic, 9 (45.0) GI	Pregabaline 1 (5.3%)	Pregabaline 2 (10.0%)	The diagnosis of CIPN was determined by a physician based on symptom history, the presence of symmetrical stocking-glove numbness, the decline in vibration sense, or paresthesia beginning after neurotoxic chemotherapy.		In the ITT analysis, the decline in activities of daily living of MHQ was significantly suppressed in the IG compared with that in the CG at T2 (difference: 7.23; 95% CI: 0.35–14.10). Similarly, in the as-treated analysis, the decline in ADL of MHQ was significantly suppressed in the IG compared with that in the CG at T2 (difference: 13.09; 95% CI: 5.68–20.49). Pain also significantly improved in the IG compared with that in the CG at T2 (difference: 13.21; 95% CI:−22.91 to−3.51).
Iravani et al. 2020 [29]	Acupuncture		19	19	57.95 (10.39)	58.79 (8.36)	7 (36.8) males, 12 (63.2) Females	8 (42.1) males, 11 (57.9) Females	Chemotherapy-induced	Chemotherapy-induced	Taxane 1 (5.3), Platinum compound 7 (36.8), Platinum compound taxane 1 (5.3), Doxorubicin/cyclophosphamide-taxane 10 (52.6)	Taxane 1 (5.3) Platinum compound 9 (47.4) Platinum compound-taxane 1 (5.3) Doxorubicin/cyclophosphamide-taxane 8 (42.1)	Breast cancer 10 (52.6) Lung cancer 1 (5.3) Ovarian cancer 0 Prostate cancer 1 (5.3) CRC 3 (15.8) Rectum 4 (21.1)	Breast cancer 8 (42.1) Lung cancer 0 Ovarian cancer 1 (5.3) Prostate cancer 1 (5.3) CRC Colon 5 (26.3) Rectum 4 (21.1)					The study revealed that acupuncture as a kind of traditional Chinese therapeutic method is significantly effective and safe in the treatment of CIPN. Moreover, acupuncture is more effective than using vitamin B1 and gabapentin as the conventional treatment.
Izgu et al. 2019 [31]	Classical massage		19	21	44.5 (10.7)	47.0 (9.6)	19 females	21 females	Chemotherapy-induced	Chemotherapy-induced	Paclitaxel	Usual care	Breast cancer; stage II 10 (52.6%); stage III 9 (47.4%)	Breast cancer stage II 6 (28.5%); stage III 15 (71.5%)			Paclitaxel-related CIPN is generally dose-dependent, and the symptoms often start in toes and fingers spreading proximally (glove and stock) Predominantly sensory axonal neuropathy, represented by sensory alterations such as paresthesia, numbness, tingling, burning sensation, and peripheral neuropathic pain. Additionally, patients may experience motor weakness, and autonomic dysfunction due to CIPN. The symptoms of CIPN may ultimately lead to debilitating limitations in routine daily living activities such as cooking, walking, driving, dressing, writing, and leisure activities.		The peripheral neuropathic pain was lower in the CMG compared to the CG at week 12 (*p* < 0.05). The sensory and motor sub-scale scores of the QOL measure showed statistically significant differences over time in favor of the CMG (*p* < 0.05). The sensory action potential amplitude of the median nerve was significantly higher, and the tibial nerve latency was significantly shorter in the CMG compared to the CG at week 12. Conclusions: This study suggested that classical massage successfully prevented chemotherapy-induced peripheral neuropathic pain, improved the QOL, and showed beneficial effects on the NCS findings.
Kneis et al. 2019 [40]	Both sets of participants engaged in endurance training, cycling at a moderate intensity for up to 30 min, staying below their IAT. In addition, the IG incorporated 30 min of balance training. The balance training sessions consisted of performing three to eight exercises, with three repetitions each, lasting 20 to 30 s per repetition. These exercises progressively escalated in difficulty by reducing the support surface, limiting visual input, incorporating motor/cognitive tasks, and inducing instability.		18	19	Median (range) 70 (44–82)	Median (range) 60 (46–75)	Male; 4 (22): female 14 (78)	Male 7 (37): female 12 (63)	Chemotherapy-induced	Chemotherapy-induced	Not mentioned	Not specified	Breast cancer 8 (44), CRC 3 (17), Gynecological cancer other than breast 2 (11), UGI cancer 1 (6), Non-small cell lung cancer 0 (0), NHL 4 (22), MM 0 (0)	Breast cancer 4 (21), CRC 10 (53), Gynecological cancer other than breast 1 (5), UGI cancer 1 (5), Non-small cell lung cancer 1 (5), NHL1 (5), MM 1 (5)	Surgery 16 (89), Radiation 8 (44), Hematopoietic cell transplantation 1 (6), Chemotherapy 18 (100)	Surgery 18 (95), Radiation 5 (26), Hematopoietic cell transplantation1 (5), Chemotherapy 19 (100)	Affected patients suffer from symptoms like pain and paraesthesia, loss of sensation and proprioception in the lower extremities resulting in muscle weakness, balance problems, and gait instability may lead to a higher risk of falling.		The CG performed better than the IG in STEO (*p* = 0.049), monopedal stance on an unstable surface (mseounstable: *p* = 0.011), Pmax_jump: *p* = 0.019, and jumping height (*p* = 0.045).
Knoerl et al. 2021 [21]	Intervention was defined as practicing ≥12 yoga sessions over the 8-week intervention period.		28	16	Median (range) 60 (33–74)	Median (range) 60 (33–74) 56.5 (40–79.0)	Male 1 (3.6%); female 27 (96.4%)	Male 1 (6.3%); female 15 (93.8%)	Chemotherapy-induced	Chemotherapy-induced	Oxaliplatin 7 (25%) Taxanes 9 (32.1%) Taxanes and platinum 12 (42.9%)	Oxaliplatin 3 (18.8%) Taxanes 4 (25%) Taxanes and platinum 9 (56.2%)	Breast 9 (32.1%) GI 7 (25%) Gynecologicalb 10 (35.7%) Multiple 2 (7.1%)	Breast 5 (31.3%) GI 3 (18.8%) Gynecologicalb 8 (50%) Multiple 0					
Knoerl et al. 2018 [26]	PROSPECT website provided cognitive-behavioral pain management techniques and information to assist people in managing pain and co-occurring symptoms following cancer treatment (such as anxiety, depression, sleep, exhaustion, and decreased cognition).		30	30	Mean age 58.93 (9.33)	Mean age 63.37 (8.36)	Male 7 (23.3%); female 23 (76.7%)	Male 8 (26.7%); female 22 (73.3%)	Chemotherapy-induced	Chemotherapy-induced	Platinum 13 (43.3) Taxanes 12 (41.4), Bortezomib 1 (3.3), Vinca alkaloids 1 (3.3), Multiple 3 (10)	Platinum 13 (43.3), Taxanes 8 (26.7), Bortezomib 0, Vinca alkaloids 2 (6.7), Multiple 7 (23.3)	Breast 13 (43.3), GI 13 (43.3), GU 0, Lung 1 (3.3), Multiple 1 (3.3), Lymphoma 2 (6.7)	Breast 10 (33.3) GI 13 (43.3)GU 1 (3.3) Lung 3 (10) Multiple 2 (6.7) Lymphoma 1 (3.3)	Neuropathic pain medications 9 (30), Other analgesics 6 (20), Neuropathic pain medications as well as other analgesics 9 (30), Antianxiety 8 (26.7), Sleep medications 3 (10), Antidepressants 4 (13.3), Fatigue medications 0	Neuropathic pain medications 6 (20) Other analgesics 8 (26.7) Neuropathic pain medications as well as other analgesics 8 (26.7) Antianxiety 8 (26.7) Sleep medications 2 (6.7) Antidepressants 5 (16.7) Fatigue medications 0	Worst pain >4		Patients with chronic painful CIPN who participated in the 8-week PROSPECT intervention reported a.94 decrease in worst pain intensity. The study’s mean reduction in pain intensity is equivalent to the effect of duloxetine, the only pharmacological medication currently indicated for the treatment of chronic painful CIPN.24 A randomized, crossover, placebo-controlled trial found that taking duloxetine 60 mg/d resulted in a 1.06 decrease in average pain intensity in people with chronic painful CIPN (*p* = 0.003, d = 0.513).54 PROSPECT, on the other hand, had no influence on the secondary outcome of average pain intensity.
Loprinzi et al. 2020 [22]	Up to five electrodes were placed in pairs in pathways of nerves that innervated—but not directly on symptomatic areas of skin. Therapy was administered to the greatest tolerable intensity. Treatments were given for 30 min on 10 consecutive weekdays.	TENS. Patients were provided with a TENS machine and instructed to use it for 30 min/day for 14 days	24	22	Median (range): 61.5 (32–82)	Median: 61 (52–75)	7 (29%)	5 (23%)	N/A	N/A	Paclitaxel: 13 Carboplatin: 7 Oxaliplatin: 4 Bortezomib: 2 Cisplatin: 2 Docetaxel: 2 Other: 3	Paclitaxel: 12 Carboplatin: 5 Oxaliplatin: 4 Bortezomib: 2 Cisplatin: 3 Docetaxel: 2 Other: 0	9 (37.5%): known disease	6 (27.2%): known disease	N/A	N/A	Pain: 16 Tingling: 8	Pain: 14 Tingling: 8	A reduction in pain/tingling scores of ≥ 50% was seen in 40% of Scrambler and 20% of TENS groups. Compared to TENS, twice as many Scrambler-treated patients had ≥ 50% improvement from baseline in pain/tingling/numbness scores. GIC scores were also improved in Scrambler compared to TENS patients for neuropathy symptoms, pain, and quality of life.
Prinsloo et al. 2017 [41]	EEG NFB for 20 sessions over a maximum of 10 weeks. Participants wore sensors on scalps and played a game that rewarded them for changing their brainwave activity. Feedback is both auditory and visual (an emotionally neutral picture appears on the screen and is accompanied by a simultaneous auditory beep when participants successfully modify their brain activity). EEG data were collected for 10 min with participants’ eyes open and with eyes closed.	Usual-care control: wait-list control where participants were offered NFB once the study was complete	35	36	62 (9.6)	63 (11)	4 (11.4%)	5 (13.9%)	N/A	N/A	Paclitaxel 31.4% Oxaliplatin 5.7 Other taxanes 34.3 Other platinum 2.9 Both taxane and platinum 14.3 Other 11.4	13.9% 5.6 38.9 8.3 11.1 22.2	Primary disease type (%): Breast 77.1 GI 2.9 Gyn 11.4 Other 8.6	Primary disease type (%): Breast 69.4 GI 8.3 Gyn 11.1 Other 11.2	N/A	N/A	Taking medication for CIPN: Yes: 28.6% No: 51.4% Missing: 20% Length of time with neuropathy (months): 24.6	Taking medication for CIPN: Yes: 30.6% No: 50% Missing: 19.4% Length of time with neuropathy (months): 25.9	The NFB group had a greater decrease in worst pain than the control (WLC) group (mean change score −2.43; WLC: 0.09). The NFB group also had decreased average pain (−2.2 NFB vs. 0.13 WLC) and pain interference (−1.86 NFB vs. −0.02 WLC). Greater decreases in 20 subscales on PQAS for NFB vs. control.
Rick et al. 2017 [36]	Magnetic field therapy. Patients used a Magcell stimulator for 5 min long therapeutic cycles twice daily (morning and evening) in frequencies from 4–12 Hz, 420 mt. Patients rested each affected palm and/or sole on the device. Patients were given one occupational therapy treatment in the affected extremity 3× a week. Treatment was for 3 months.	Placebo	21	23	Median (range): 58. (28–73)	Median (range): 58 (43–73)	6 (29%)	7 (30%)	N/A	N/A	Platinum: 10 (48%) Taxane: 10 (48%) Vinca alkaloids: 3 (14%) Other: 9 (43%)	Platinum: 6 (26%) Taxane: 11 (48%) Vinca alkaloids: 3 (13%) Other: 8 (35%)	Lymphoma: 4, 19% Breast: 7, 33% Ovarian: 4, 19% CRC: 5, 24% Other: 1, 5%	Lymphoma: 2, 9% Breast: 11, 48% Ovarian: 2, 9% CRC: 5, 22% Other: 3, 12%	N/A	N/A	N/A	N/A	Significantly reduced sensory neurography for MFT vs. placebo at the end of the study (3 months) (on the peroneal nerve) and between enrollment and 3 weeks into the study (on the ulnar nerve). There was no statistically significant difference between the two groups in pain detection end score.
Schwenk et al. 2016 [44]	Balance training technology was delivered over a computer with five inertial sensors (mounted on the shank, thigh, and lower back). Sensor data were transmitted at 100 Hz to visualize ankle and knee movements. Participants were given audio/visual rewards or notifications of error at the end of each task. Participants attended two 45 min sessions per week for 4 weeks. Sessions were as follows: ankle point-to-point reaching, and virtual obstacle course crossing. Balance exercises included leaning and dynamic weight shifting to improve postural balance. Measurements were taken using wearable sensors.	No intervention but recommended to perform regular exercise at home	11	11	68.73 +/− 8.72	71.82 +/− 8.85	4 (36.4%)	5, (45.6%)	N/A	N/A	Unspecified	Unspecified	Unspecified	Unspecified	N/A	N/A	Numbness, NRS score 0–10 (mean, SD): 5.9 +/− 3.25 Pain, NRS score 0–10 (mean, SD): 3.10 +/− 3.31	Numbness, NRS score 0–10 (mean, SD): 4.91 +/− 3.08 Pain, NRS score 0–10 (mean, SD): 2.44 +/− 2.83	The sway of the hip, ankle, and center of mass was significantly reduced in the intervention vs. control group while standing in feet close position with eyes open (except AP center of mass sway) and semi-tandem position (except ankle sway). No significant improvement in balance with eyes closed, gait speed, or fear of falling in the intervention group.
Song et al. 2020 [34]	Low-frequency electrical stimulation (ES) wristband device used at 100 ua, 40 Hz. Participants used the device at least 2× a day for ≥ 120 min, including an uninterrupted 1 h of use, for more than 14 days. Electrical stimulation was applied at the unilateral PC6 acupoint (three finger breadths above the wrist crease between the palmaris longus and flexor carpi radialis tendons). Participants also took duloxetine or pregabalin.	Placebo device (SES) for the same duration with the same appearance and labeling. Participants also took duloxetine or pregabalin.	36	36	49.91 (8.85)	49.71 (8.24)	0%	0%	N/A	N/A	TAC (docetaxel + doxorubicin + cyclophosphamide): 1 (3%)AC (doxorubicin + cyclophosphamide) + mt(docetaxel): 11 (31%)AC (doxorubicin + cyclophosphamide) + paclitaxel: 5 (14%)AC (doxorubicin + cyclophosphamide): 1 (3%)TA (docetaxel + doxorubicin): 2 (6%) TC (docetaxel + cyclophosphamide): 12 (33%) CMF (cyclophosphamide + methotrexate + 5-FU): 4 (11%)	TAC (docetaxel + doxorubicin + cyclophosphamide): 2 (6%)AC (doxorubicin + cyclophosphamide) + mt(docetaxel): 14 (39%)AC (doxorubicin + cyclophosphamide) + paclitaxel: 2 (6%)AC (doxorubicin + cyclophosphamide): 1 (3%)TA (docetaxel + doxorubicin): 5 (14%)TC (docetaxel + cyclophosphamide): 11 (31%)CMF (cyclophosphamide + methotrexate + 5-FU): 1 (3%)	Breast cancer, 100%	Breast cancer, 100%	N/A	N/A	36 (100%)	36 (100%)	No difference in the intensity of CIPN on NRS between ES and SES (CG) groups, but in both groups, the intensity of CIPN sx was reduced from baseline. For participants with high NRS (n = 6), significant results were seen in both ES and SES arms. No changes from baseline for TNS, EORTC-QLQ CIPN20, or FACT-B; however, the mean change from baseline was greater in ES than SES. No improvement in pain-related symptoms. Significant differences between ES and SES were seen only with pts with cold arthralgia under IPIE-CIPN.
Streckmann et al. 2014 [33]	IG had exercise twice a week for 1 h each for 36 weeks under supervision of certified personnel; only interrupted for 24 hrs after administration of chemo. Sessions consisted of: 10–30 min ride at 60–70% max HR on bicycle dynamometer or treadmill walk; four postural stabilization tasks of progressive difficulty and surface instability–three sets at 20 s intervals with 20 s rest between each set and 1 min between exercises; and four resistance exercises for 1 min at maximum force, for inpatients substituted with a thera-band.	Control, 36 weeks. Patients still underwent chemo.	28	28	Age, range: 44 (20–67)	48 (19–73)	20 (71%)	22 (79%)	N/A	N/A	N/A	N/A	Hodgkin’s: 7 (25%) B-NHL: 13 (46%) T-NHL: 3 (11%) Multiple myeloma: 5 (18%)	Hodgkin’s: 5 (18%) B-NHL: 13 (46%) T-NHL: 3 (11%) Multiple myeloma: 8 (29%)	N/A	N/A	5 (25%)	4 (24%)	IG significantly improved quality of life (only in the first 12 weeks), constipation, diarrhea, and pain. Average peripheral deep sensitivity (PNP) incidence was lower in IG than in CG (12% vs. 27%). PNP symptoms decreased in 87.5% of IG and 0% in CG once developed. At 36 weeks, the number of patients with PNP was significantly lower in IG. IG increased activity level by 2.5 MET/week on average, while CG activity level deteriorated. Balance control was significantly different in IG compared to CG.
Streckmann et al. 2019 [32]	Training twice a week for 6 weeks with supervision. Two intervention arms: SMT and WBV Sensorimotor training (SMT): progressively more difficult balancing exercises on progressively unstable surfaces. Four exercises per session; each exercise done 3× for 20 s with a 40 s rest between each set and 1 min rest between each exercise. Whole body vibration training (WBV): on a side-alternating vibration platform wearing anti-slip socks or shoes. Four progressing sets of 30–60 s vibration exercises with frequency from 18–35 Hz and amplitude of 2–4 mm with one minute rest between exercises. They had previously performed endurance and strength training (standardized protocol, equipment-based circuit, twice a week for 45 min at moderate intensity) for the past 6 months to 2 years with no self-reported effect on the neuropathy. They had previously performed endurance and strength training (standardized protocol, equipment-based circuit, twice a week for 45 min at moderate intensity) for the past 6 months to 2 years with no self-reported effect on the neuropathy.	Two groups: Oncologic control group (OCG) Healthy control group (HCG) Duration unspecified	SMT: 10 WBV: 10	CG: 10 HCG: 10	Average (Range) SMT: 56 (47–74) WBV: 59 (51–69)	Average (range) CG: 59 (49–70) HCG: 57 (47–68)	SMT: 4 (40%) WBV: 2 (20%)	CG: 3 (30%) HCG: 4 (40%)	N/A	N/A	SMT (n) Taxane: 6 Platinum derivate: 5 Vinca Alklaloid: 1 WBV (n) Taxane: 5 Platinum derivate: 2 Vinca Alklaloid: 1	GC (n) Taxane: 4 Platinum derivate: 4 Vinca alkaloid: 2 HGC (n) Taxane: 0 Platinum derivate: 0 Vinca alkaloid: 0	SMT: Mamma-CA: 4 Ovarian-CA: 2 Adeno-CA: 1 Colon-CA: 1 Pancreatic-CA: 0 T cell NHL: 1 M. Hodgkin: 0 Plasmocytoma: 0 MM: 0 Rectal-CA: 1 Lung-CA: 0 WBV: Mamma-CA: 4 Ovarian-CA: 0 Adeno-CA: 1 Colon-CA: 0 Pancreatic-CA: 1 T cell NHL: 0 M. Hodgkin: 2 Plasmocytoma: 1 MM: 1 Rectal-CA: 0 Lung-CA: 0	CG: Mamma-CA: 4 Ovarian-CA: 1 Adeno-CA: 1 Colon-CA: 1 Pancreatic-CA: 0 T cell NHL: 0 M. Hodgkin: 0 Plasmocytoma: 1 MM: 0 Rectal-CA: 1 Lung-CA: 1 HCG: 0	N/A	N/A	SMT Duration of neuropathy, years (range): 2 (1–5) Incidence of neuropathy after cycles of chemo, cycle (range): 3 (1–8) Therapy reduced/terminated due to neuropathy (n,%): 3 (30%) WBV: Duration of neuropathy, years (range): 2 (1–5) Incidence of neuropathy after cycles of chemo, cycle (range): 3 (1–10) Therapy reduced/terminated due to neuropathy (n,%): 3 (30%)	CG Duration of neuropathy, years (range): 2 (1–5) Incidence of neuropathy after cycles of chemo, cycle (range): 3 (1–10) Therapy reduced/terminated due to neuropathy (n,%): 3 (30%)	Significant intergroup differences were found for Achilles and patellar tendon reflexes, with the SMT group having improved outcomes (SMT vs. CG P = 0.020, d = 1.514) and Achilles tendon reflex (SMT vs. CG P = 0.042, d = 0.978/SMT vs. WBV P = 0.036, d = 0.978). For pain and dyspnea, the WBV group had improved outcomes (pain: F(2,25) = 3.278, P = 0.054, dyspnea F(2,25) = 3.294, P = 0.054).
Waibel et al. 2021 [37]	Balance training was added to moderate endurance training. The one-on-one training sessions took place 2×/week over 12 weeks (30 min moderate endurance + 30 min balance training). Balance exercise sessions included 3–8 exercises with three repetitions of 20–30 s each, involving progressively increasing exercise difficulty by reducing the support surface and visual input, adding motor/cognitive tasks, and inducing instability.	Moderate endurance training (12 weeks). The one-on-one training sessions took place 2×/Week over 12 weeks (30 min each)	16	15	67 (44–82)	60 (46–75)	3 (19)	6 (40)	CIPN symptoms after having completed anti-tumor treatment	CIPN symptoms after having completed anti-tumor treatment	N/A	NA	Multiple (colorectal, breast, gynecological, upper GI, NHL)	Multiple (colorectal, breast, gynecological, upper GI, NHL)	N/A	N/A	Patients reporting chemotherapy-induced peripheral neuropathy	Patients reporting chemotherapy-induced peripheral neuropathy	Patients reporting CIPN
Zhi et al. 2021 [25]	The yoga group practiced daily for 60 min for 8 weeks via video alongside in-person group classes twice a week. The yoga protocol emphasized breathwork (pranayama) to regulate the autonomic nervous system and modifiable postures (asanas) to improve musculoskeletal flexibility, strength, and balance.	The wait-list usual care control arm did not receive interventions throughout the 12 weeks.	21	20	60.0 (35.5, 77.9)	62.3 (42.4, 79.0)	0 (0)	0 (0)	Completed neurotoxic chemotherapy (eg, paclitaxel, docetaxel, carboplatin) at least 3 months before enrollment	Completed neurotoxic chemotherapy (eg, paclitaxel, docetaxel, carboplatin) at least 3 months before enrollment	Neurotoxic chemotherapy (eg, paclitaxel, docetaxel, carboplatin)	Neurotoxic chemotherapy (eg, paclitaxel, docetaxel, carboplatin)	Breast and gynecological cancer stage I–III	Breast and gynecological cancer stage I–III	NA	NA	Reported moderate-to-severe CIPN, defined as tingling, numbness, or pain rated ≥4 on the 11-point NRS; and maintained an ECOG performance status of 0–2.	Reported moderate-to-severe CIPN, defined as tingling, numbness, or pain rated ≥4 on the 11-point NRS; and maintained an ECOG performance status of 0–2.	At week 8, mean NRS pain decreased by 1.95 points (95% confidence interval [CI] = −3.20 to −0.70) in yoga vs. 0.65 (95% CI = −1.81 to 0.51) in usual care (P = 0.14). FACT/GOG-Ntx improved by 4.25 (95% CI = 2.29 to 6.20) in yoga vs. 1.36 (95% CI = −0.47 to 3.19) in usual care (P = 0.035). Functional reach, an objective functional measure predicting the risk of falls, improved by 7.14 cm (95% CI = 3.68 to 10.59) in yoga and decreased by 1.65 cm (95% CI = −5.00 to 1.72) in usual care (P = 0.001). Four grade 1 adverse events were observed in the yoga arm.
Zimmer et al. 2018 [38]	Eight-week supervised exercise program, including endurance, resistance, and balance training (2×/week for 60 min).	CG received written standard recommendations to obtain physical fitness.	17	13	68.53 (50–81)	70.00 (50–81)	(female/male), n (%) 5 (29.4)/12 (70.6)	(female/male), n (%) 4 (30.8)/9 (69.2)	Chemotherapy-induced	Chemotherapy-induced	Oxaliplatin	Oxaliplatin	Colorectal cancer	Colorectal cancer	Capecitabine + antibody, FOLFOX ± antibody, FOLFIRI ± antibody, -5-fu/folinic acid ± antibody	Capecitabine + antibody, FOLFOX ± antibody, FOLFIRI ± antibody, -5-fu/folinic acid ± antibody			
Stuecher et al. 2019 [45]	The intervention comprised 12 weeks of home-based walking exercise. In line with cancer-specific guidelines [9], the aim was to complete 150 min of moderate-intensity walking per week. Moderate intensity was regulated via Borg’s self-rating of perceived exertion (RPE).	Wait-list CG participants received usual care dependent on the hospital guidelines as well as oncologists’ and physicians’ considerations. The CG also received weekly phone calls and were asked about their wellbeing	13	15	66.8 ± 7.8	65.9 ± 7.9	Male 8 (61.5%); female 5 (38.5%)	Male 8 (53.3%); female 7 (46.7%)	Chemotherapy-induced	Chemotherapy-induced	NA	NA	GI cancer	GI cancer	Palliative, neoadjuvant, adjuvant	Palliative, neoadjuvant, adjuvant			
Vollmers et al. 2018 [42]	Regular physical training and sensorimotor exercises 2×/week throughout the Paclitaxel chemotherapy treatment and for 6 further weeks after chemotherapy termination	CG (n = 19) received an instruction sheet informing them about the current state of science concerning physical activity in malignant diseases and suggesting a regular physical activity designed autonomously by the patients	17	19	48.56 (±11.94)	52.39 (±10.14)	All females	All females	Chemotherapy-induced	Chemotherapy-induced	Paclitaxel	Paclitaxel	Breast cancer	Breast cancer	Sensorimotor exercise				

P = physical intervention; C = comparator; * = full description with duration of intervention; 5-FU = 5-fluorouracil; CRC = colorectal cancer; UGI = upper gastrointestinal; GI = gastrointestinal; GU = genitourinary; NHL = non-Hodgkin’s lymphoma; MM = multiple myeloma; IAT = individual anaerobic threshold; US = ultrasound; IG = intervention group; CG = control group; ITT: intention-to-treat; STEO = semi-tandem stance with eyes open; Pmax_jump = maximal jumping power; TENS = transcutaneous electrical nerve stimulation; EEG = electroencephalogram; NFB = neurofeedback; NRS = numerical rating scale; ECOG = Eastern Cooperative Oncology Group3.2. Participant Demographics. Among the included studies, the age of participants varied widely, with the youngest average being 44 years old [33] and the oldest reaching an average of 72 years [30]. This age range demonstrates the broad applicability of the interventions across a diverse adult age spectrum. Additionally, the total number of participants involved in these studies was quite substantial, encompassing a range from as few as 10 individuals [27] to as many as 168 in larger studies [28]. Overall, these studies collectively involved a total of 1167 participants, providing a robust dataset for analyzing the effectiveness of various interventions.

## Data Availability

Data sharing is not applicable. No new data were created or analyzed in this study.

## Supplementary Materials

The following supporting information can be downloaded at: https://www.mdpi.com/article/10.3390/healthcare13131526/s1. Table S1: PRISMA Checklist; Table S2: Literature search keywords; Table S3: Eligibility criteria for studies upon screening the search results obtained.
